# Recurrent small bowel obstruction caused by Burkitt lymphoma in an elderly man: a case report and review of the literature 

**DOI:** 10.1186/s13256-020-02449-y

**Published:** 2020-08-12

**Authors:** Saro Kasparian, Ethan Burns, Ahmed Shehabeldin, Melina Awar, Sai Ravi Pingali

**Affiliations:** 1 Department of Internal Medicine, Houston Methodist Hospital, Houston, TX 77030 USA; 2 Department of Pathology, Houston Methodist Hospital, Houston, TX 77030 USA; 3 Division of Hematology and Oncology, Houston Methodist Hospital, Houston, TX 77030 USA

**Keywords:** Sporadic Burkitt lymphoma, Small bowel obstruction, Recurrent

## Abstract

**Background:**

Acute small bowel obstruction is a common surgical emergency usually caused by abdominal adhesions, followed by intraluminal tumors from metastatic disease. Although lymphomas have been known to cause bowel obstruction, Burkitt lymphoma is seldom reported to induce an obstruction in the adult population.

**Case presentation:**

A 78-year-old Hispanic man with a history of abdominal interventions presented to our hospital with abdominal pain. Computed tomography revealed a partial small bowel obstruction attributed to local inflammation or adhesions. Medical management with bowel rest and nasogastric decompression resulted in resolution of symptoms and quick discharge. He returned 2 days later with worsening abdominal pain. Repeat imaging showed progression of the partial small bowel obstruction, but with an additional 1.6-cm nodular density abutting the anterior aspect of the gastric antrum and lobulated anterior gastric antral wall thickening. He was taken to the operating room, where several masses were found. Intraoperative frozen sections were consistent with lymphoma, and pathology later revealed Burkitt lymphoma. Disease was found on both sides of the diaphragm by positron emission tomography. After the initial resection and adjuvant chemotherapy, the patient is alive and well about 14 months after resection.

**Conclusions:**

Small bowel obstruction is uncommonly due to Burkitt lymphoma in the geriatric population and is more frequently seen in the pediatric and young adult populations. Burkitt lymphoma is very aggressive with rapid cell turnover leading to significant morbidity. The rapid recurrence of an acute abdominal process should prompt an investigation for a more sinister cause such as malignancy.

## Introduction

Acute small bowel obstruction (SBO) is a common surgical emergency. Approximately 300,000 patients are hospitalized annually for mechanical SBO [1, 2], and this comprises 12–16% of hospital admissions for abdominal pain in the United States. The most significant risk factors for an SBO are abdominal adhesions due to prior abdominal surgeries [3]. Intraluminal tumors are the second most common cause of SBO, the majority of which are due to metastatic disease [4]. Lymphomas make up an estimated 24% of neoplasia-induced bowel obstruction [5], with few cases attributed to Burkitt lymphoma (BL) in the adult population. The present case report highlights the rare presentation and clinical implications of recurrent SBO due to sporadic BL.

## Case presentation

**Fig. 1:**
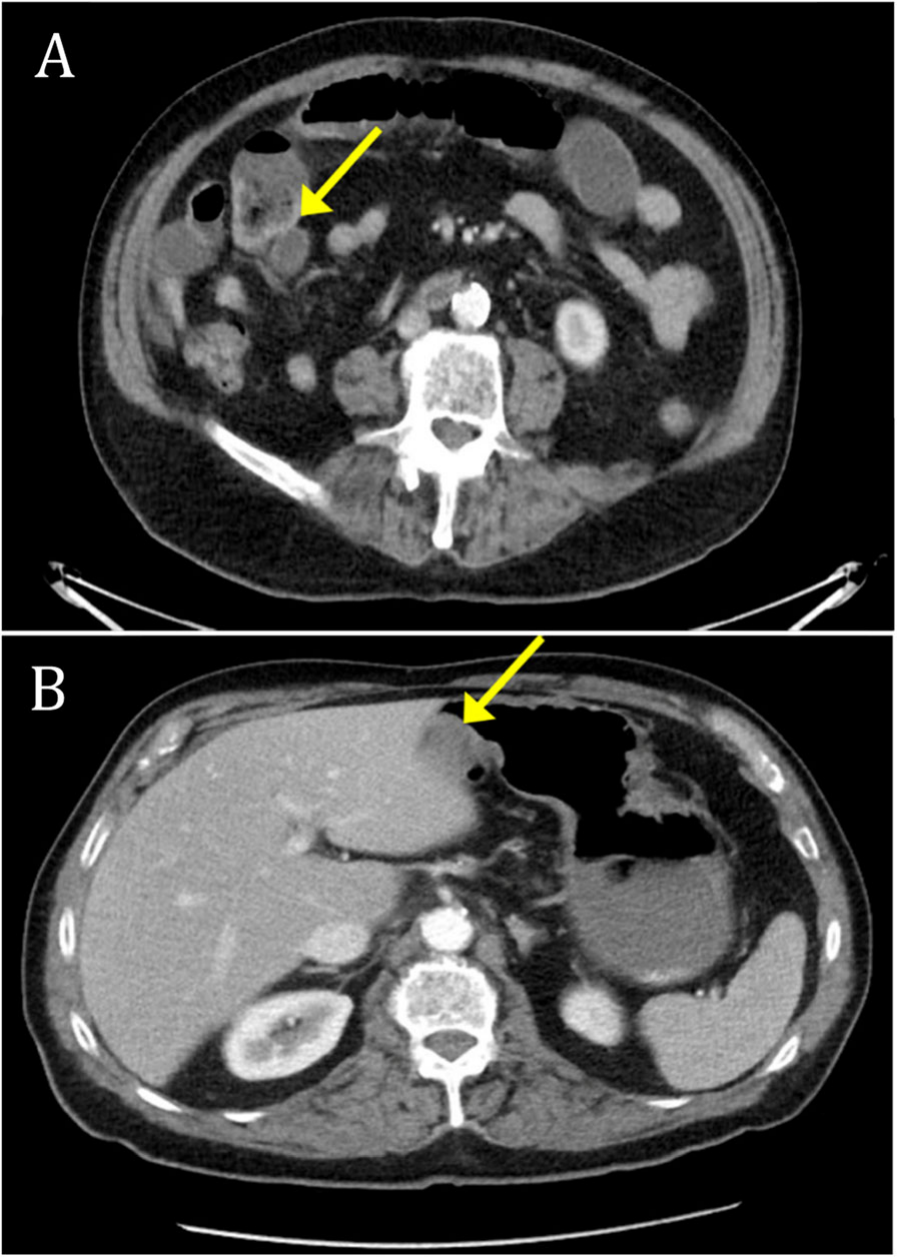
Computed tomography (CT) of the abdomen. Abdominal CT scans of first admission compared with second admission. **a** Partial bowel obstruction noted on first admission with transition point (*arrow*). **b** Nodular mural thickening of the anterior aspect of the gastric antrum (*arrow*)

A 78-year-old Hispanic man presented to the emergency department (ED) of our hospital with abdominal pain accompanied by nausea and bilious emesis. His history was significant for an open appendectomy, laparoscopic cholecystectomy, and umbilical hernia repair. He had no significant family history and did not smoke, drink alcohol, or use illicit substances. The patient denied fever or night sweats but complained of anorexia and unintentional weight loss in the month leading up to admission. His physical examination revealed normal bowel sounds, no tympani on percussion, no palpable masses, no hepatosplenomegaly, no fluid wave, and a soft but diffusely tender and distended abdomen. Computed tomography (CT) of his abdomen and pelvis with oral and intravenous contrast revealed small bowel distention and a short segment of bowel wall thickening with enhancement in the right lower quadrant consistent with a partial SBO, likely arising from either local inflammation or adhesion (Fig. 1a). He was treated medically with bowel rest and decompression with nasogastric tube placement. The patient reported significant relief shortly after decompression, with complete resolution within 48 hours. His diet was advanced, and he was discharged to home with close outpatient follow-up.

He returned to our ED 2 days after discharge with recurrence of abdominal pain, distention, nausea, and bilious vomiting. Repeat CT of his abdomen and pelvis with oral and intravenous contrast showed a moderate increase in the small bowel distention with a transition point in the left middle abdomen consistent with moderate progression of the partial SBO. CT also revealed a 1.6-cm nodular density abutting the anterior aspect of the gastric antrum and lobulated anterior gastric antral wall thickening concerning for atypical gastritis or a gastric tumor (Fig. 1b). Because of an unsuccessful trial of decompression, the patient was taken to the operating room for adhesiolysis and possible bowel resection.

**Fig. 2:**
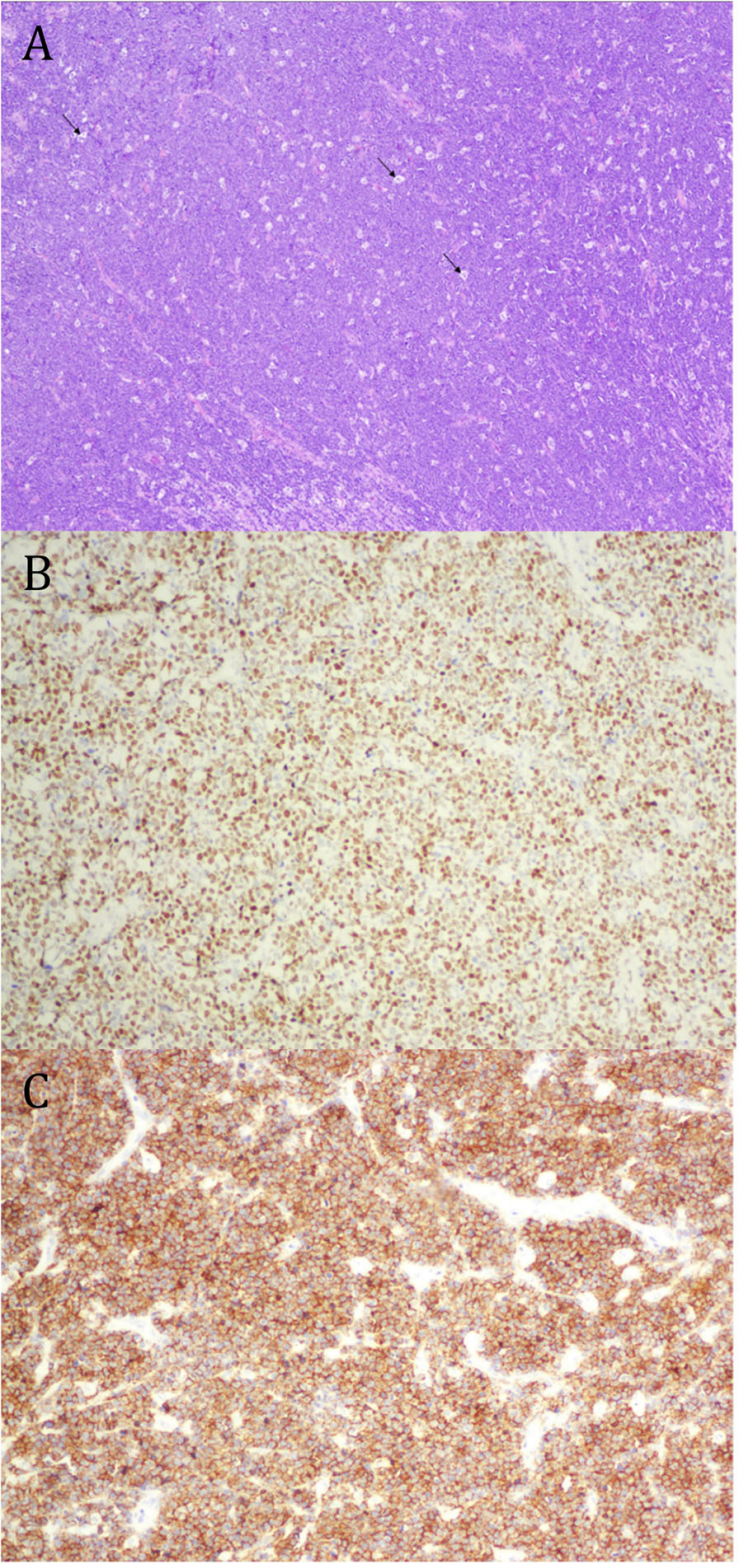
Frozen sections and microscopic analysis of intraoperative findings. **a** “Starry sky” appearance consisting of sheets of intermediate-sized lymphocytes represent the “dark sky.” The intervening dispersed histiocytes with debris (tingible body macrophages) represent the “stars” (*arrows*). **b** c-Myc stain showing a nuclear pattern. **c** CD20 stain showing a membranous pattern

**Fig. 3:**
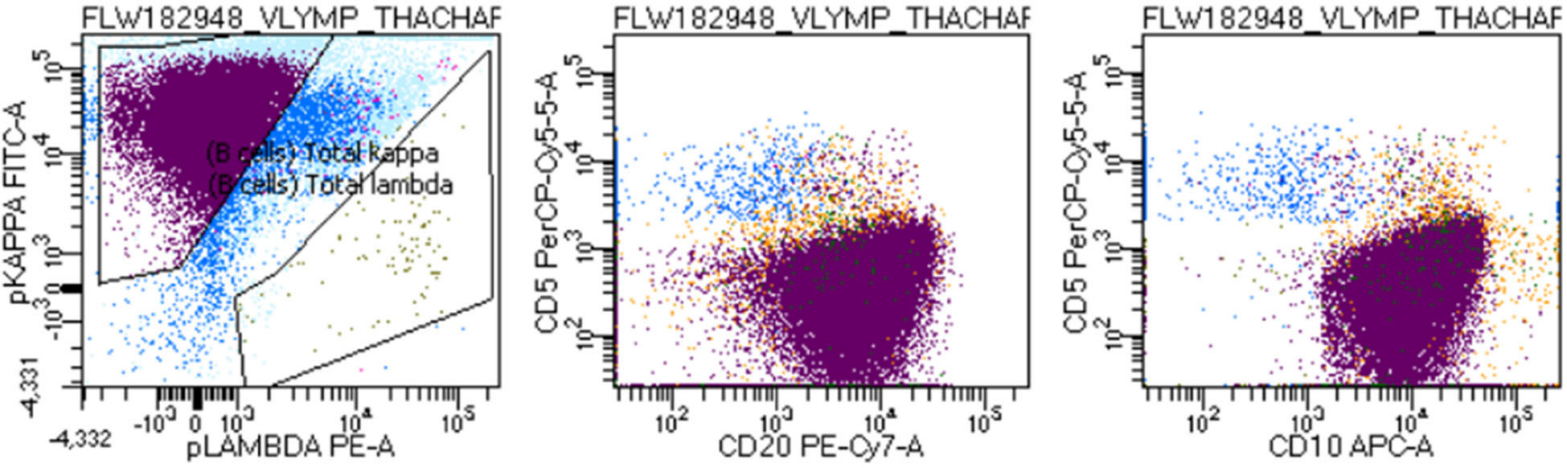
Flow cytometry. Results of flow cytometry consistent with B-cell lymphoma, which is kappa-light chain restricted with expression of CD10 and lack of CD5 expression

**Fig. 4:**
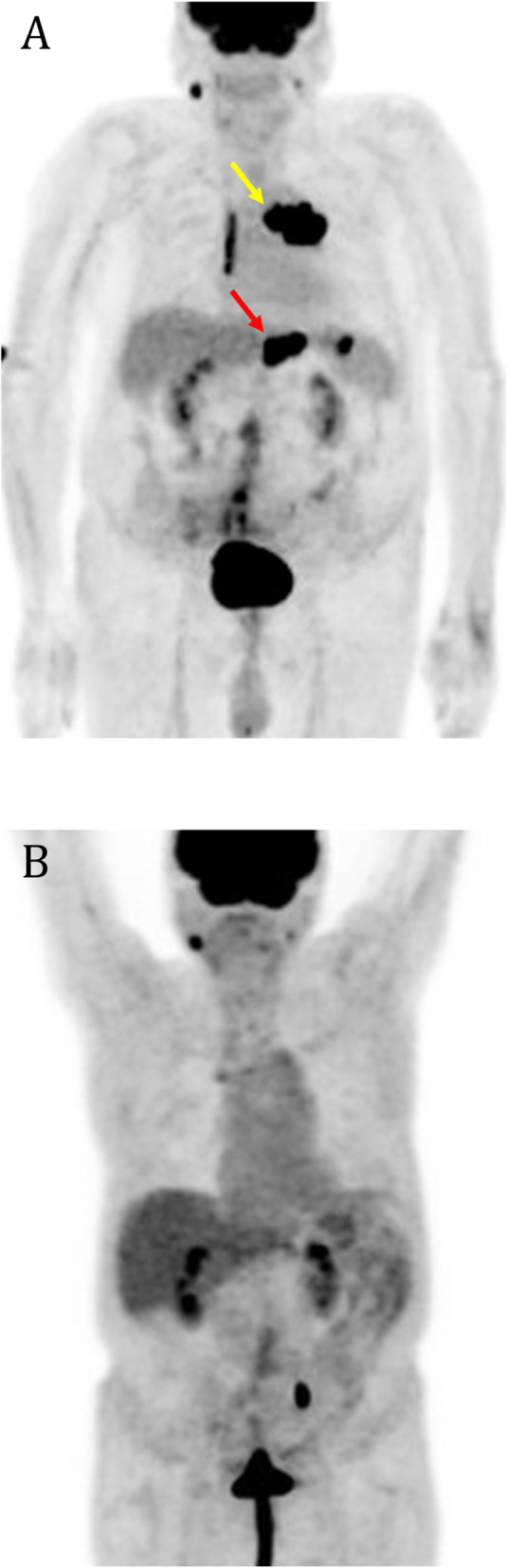
Positron emission tomography (PET) scan. **a** Pretreatment PET with active lymphoma on both sides of the diaphragm, including chest adenopathy and multifocal gastric involvement in the abdomen. *Yellow arrow*: chest adenopathy with standardized uptake value (SUV) of 20.4. *Red arrow*: gastric antrum uptake with SUV of 14.1. **b** Post-treatment PET about 6 months after resection showing no definite evidence of lymphoma

Intraoperative findings included a completely obstructive mass within the ileum, a partial obstruction by two masses within the jejunum, and a nonobstructive gastric antral mass. He underwent two small bowel resections with excision of the small bowel tumors. Intraoperative frozen section analysis of the small bowel tumors was consistent with lymphoma. The tissue sample was positive for cluster of differentiation (CD)20, paired box protein 5 (PAX5), B-cell lymphoma protein (BCL)-6, and cellular myelocytomatosis (c-Myc), and it was negative for BCL-2, CD3, and CD5 (Fig. 2). The result of Epstein-Barr virus polymerase chain reaction testing was also positive. Flow cytometry showed an abnormal B-cell population positive for CD10, CD19, CD20, CD22, CD38, and CD45 with kappa-light-chain restriction, but it was negative for T-cell markers, CD5, and lambda light chain (Fig. 3). The final pathology was consistent with BL. He underwent a bone marrow biopsy and aspirate that was negative for lymphoma. Positron emission tomography (PET) demonstrated active disease on both sides of the diaphragm, including adenopathy in the chest, gastric antrum, and the greater gastric curvature, consistent with a stage III BL (Fig. 4a).

After postoperative recovery, the patient was initiated on six cycles of adjuvant combination chemotherapy with dose-adjusted etoposide, prednisone, vincristine, cyclophosphamide, hydroxydaunorubicin, and rituximab (DA-EPOCHR), as well as intrathecal (IT) methotrexate for central nervous system (CNS) prophylaxis. Overall, he tolerated the chemotherapy regimen well, except for a subsequent SBO during the sixth cycle that was managed conservatively. A post-treatment PET scan showed complete remission (Fig. 4b), and the patient is still being followed 14 months after initial resection.

## Discussion

We present a unique case of a recurrent SBO due to BL in an elderly man, an uncommon presentation of an uncommon diagnosis in the elderly. BL cells are mature B-cells arising from postgerminal centers of lymph nodes. They generally have a high proliferative index (Ki67) approaching 100%, and their typical immunophenotype expresses CD19, CD20, CD22, CD45, CD79, and germinal center markers CD10 and BCL6 [6]. Their high rate of proliferation is mediated by the activation of the MYC oncogene on chromosome 8 via translocation, most commonly to the immunoglobulin heavy chain locus found on chromosome 14 t(8:14) [7]. The translocation is thought to occur during immunoglobulin class switching and somatic hypermutation in B-cells causing double-stranded DNA breaks [8]. This leads to a constitutive overexpression of the MYC protein, resulting in unregulated cell proliferation. MYC is a transcription factor involved in regulating approximately 15% of all genes, particularly those involved in cell growth [9]. On microscopic examination, these mutations lead to the development of sheets of intermediate-sized lymphocytes and dispersed histiocytes, classically referred to as the “starry sky” pattern (Fig. 2).

BL is a rare, highly aggressive, and rapidly dividing form of non-Hodgkin lymphoma (NHL) that accounts for < 1% of adult NHL in the United States [10]. Three forms of BL have been recognized: the endemic form, the immunodeficiency-associated form, and the sporadic form. The endemic form comprises 30–50% of childhood cancers in equatorial Africa, with an approximate incidence of 3–6 cases per 100,000 children per year [11]. The immunodeficiency-associated variant is seen primarily in individuals with HIV infection counterintuitively with higher CD4 counts, and generally in the absence of opportunistic infections [12].

The sporadic form is commonly encountered in the United States and Europe, comprising 30% of pediatric lymphomas, with a peak incidence at age 11 years [10]. In adults, BL is most prevalent in Caucasian males with a median age of 30 years and a male predominance [13, 14]. Sporadic BL often presents with bulky tumors in extranodal sites, frequently in the abdomen [15], but it is also seen in the kidney, pancreas, liver, spleen, breast, and ovaries and rarely the head and neck [15–17]. Symptoms of BL in the gastrointestinal tract can be nonspecific and include abdominal pain, nausea, and vomiting or can be more pronounced in the form of obstruction, acute appendicitis, or intussusception. These abdominal syndromes are more frequently reported in young adult and pediatric patients [18, 19]. A review of the available literature suggests that abdominal syndromes as the initial presentation of BL are seldom reported in the elderly patient population. Furthermore, the presence of recurrent abdominal syndromes such as SBO as the initial presentation has not been reported outside of the young adult and pediatric populations (Table 1). To our knowledge, this is the first reported case of BL presenting as a recurrent SBO.

**Table 1:** Literature review of adult Burkitt lymphoma with obstructive symptoms

Author	Age (yr)	Sex	BL type	Presentation	Abdominal syndrome	Syndrome recurrence	Treatment regimen	Outcome
Fernandes *et al*. [20]	21	Female	Sporadic	Abdominal pain	Intussusception	Yes	Resection alone	NR
Simson *et al.* [21]	22	Female	Sporadic	Persistent abdominal pain, appendicitis	Appendicitis then intussusception	No	Resection with adjuvant chemotherapy	NR
Sharma *et al*. [22]	26	Male	HIV	Viral syndrome, abdominal distention	Obstruction	No	Resection then R-CODOX-M/IVAC	NR
Wetter *et al*. [23]	29	Female	HIV	Abdominal pain	Intussusception	No	Resection	NR
Gupta *et al*. [24]	33	Female	HIV	Loose stool, abdominal discomfort, nausea, obstructive jaundice	Obstructive jaundice	No	CODOX-M/IVAC	Complete remission
Felix *et al*. [25]	34	Male	NR	Abdominal pain	Intussusception	Yes	Resection	NR
Özant *et al*. [26]	37	Female	Sporadic	Abdominal pain, nausea, vomiting	Obstruction due to intussusception	No	Resection with hyperCVAD	Complete remission
Mizutani *et al*. [27]	38	Male	NR	Abdominal pain	Obstruction	No	Cyclophosphamide and doxorubicin with R-CODOX-M/IVAC	Complete remission
Zerwas *et al*. [28]	52	Male	NR	Abdominal pain, nausea, vomiting	Intussusception	NR	Resection	NR
Kasparian *et al.* [this report]	78	Male	Sporadic	Abdominal pain, nausea, vomiting	Obstruction	Yes	Resection then DA-EPOCHR	Complete remission

This is the first reported case of BL presenting with recurrent SBO in the geriatric population

*Abbreviations: DA-EPOCHR* Dose-adjusted etoposide, prednisone, vincristine, cyclophosphamide, hydroxydaunorubicin, and rituximab, *HIV* Human immunodeficiency virus, *hyperCVAD* Cyclophosphamide, vincristine, doxorubicin, *NR* Not reported, *(R) CODOX-M /IVAC* (Rituximab) cyclophosphamide, vincristine, doxorubicin, and high-dose methotrexate, with intrathecal methotrexate, alternating with ifosfamide, etoposide, and cytarabine-A

The prognosis for BL in the era of chemoimmunotherapy is generally age-dependent. Analysis of the Surveillance, Epidemiology, and End Results Program database showed that among those diagnosed between 2002 and 2008, 5-year survival decreased, depending on age, from 87%, 60%, 48%, to 33% for ages < 20 years, 20–39 years, 40–59 years, and > 59 years, respectively [29]. In addition, poorer outcomes were seen in black patients and those with advanced disease [29].

BL is an aggressive cancer and requires prompt recognition and treatment to improve outcomes. Consensus on first-line therapy for BL is lacking, but combination chemotherapy along with CNS chemoprophylaxis is recommended. Ann Arbor or Murphy scales are used for staging, which is further classified as low or high risk on the basis of the bulk of disease, number of sites, and lactate dehydrogenase elevation [12]. The classic Magrath method [11] uses cyclophosphamide, vincristine, doxorubicin, and high-dose methotrexate, with IT methotrexate, alternating with ifosfamide, etoposide, and cytarabine-A (CODOX-M/IVAC) [30]. The modified Magrath method is preferred in older adults (median age 47 years) due to its better-tolerated toxicity profile, with 86% of patients achieving a complete response and 64% an event-free survival at 29 months [30]. In a phase II study, Evens *et al.* reported that the addition of rituximab combined with liposomal doxorubicin achieved an overall response rate of 100% with complete remission in 92%. At 34-month median follow-up, the 2-year progression-free survival (PFS) and overall survival (OS) rates for all patients were 80% and 84%, respectively (low-risk, both 100%; high-risk, 76% and 81%, respectively) [31]. Alternative first-line regimens recommended by the National Comprehensive Cancer Network include DA-EPOCHR and cyclophosphamide, vincristine, and doxorubicin (HyperCVAD). The PFS and OS in the EPOCH group were found to be 95% and 100%, respectively, at median follow-up of 86 months [32], whereas the 36-month OS and PFS in the HyperCVAD group were 88% and 89%, respectively [33]. CNS involvement is common in NHL, and most regimens employ some form of CNS prophylaxis, usually in the form of IT methotrexate and/or cytarabine, to prevent future CNS relapse [34]. Surgery and radiation therapy do not have routine roles in treatment of BL other than in the intervention of emergent abdominal syndromes.

## Conclusions

SBOs are uncommonly due to sporadic BL in the adult populace. BL is seldom diagnosed in the geriatric population. This case illustrates the rapid growth and significant morbidity of sporadic BL presenting as recurrent SBO. The unexpected recurrence of an abdominal disease process should lead to the consideration of more aggressive pathologies such as intra-abdominal malignancies.

## Data Availability

Not applicable.

## Abbreviations

BCL: B-cell lymphoma
BL: Burkitt lymphoma
CD: Cluster of differentiation
c-Myc: Cellular myelocytomatosis
CNS: Central nervous system
CODOX-M/IVAC: Cyclophosphamide, vincristine, doxorubicin, high-dose methotrexate, ifosfamide, etoposide, and cytarabine-A
CT: Computed tomography
DA-EPOCHR: Dose-adjusted etoposide, prednisone, vincristine, cyclophosphamide, hydroxydaunorubicin, and rituximab
ED: Emergency department
HyperCVAD: Cyclophosphamide, vincristine, doxorubicin
IT: Intrathecal
NHL: Non-Hodgkin lymphoma
OS: Overall survival
PAX 5: Paired box protein 5
PET: Positron emission tomography
PFS: Progression-free survival
SBO: Small bowel obstruction
